# Surveillance and Genome Analysis of Human Bocavirus in Patients with Respiratory Infection in Guangzhou, China

**DOI:** 10.1371/journal.pone.0044876

**Published:** 2012-09-11

**Authors:** Lin Xu, Xia He, Ding-mei Zhang, Fa-shen Feng, Zhu Wang, Lin-lin Guan, Jue-heng Wu, Rong Zhou, Bo-jian Zheng, Kwok-yung Yuen, Meng-feng Li, Kai-yuan Cao

**Affiliations:** 1 Department of Microbiology, Zhongshan School of Medicine, Sun Yat-sen University, Guangzhou, People's Republic of China; 2 Key Laboratory of Tropical Disease Control, Ministry of Education, Sun Yat-Sen University, Guangzhou, People's Republic of China; 3 Sun Yat-sen University – University of Hong Kong Joint Laboratory of Infectious Disease Surveillance, Sun Yat-sen University, Guangzhou, People's Republic of China; 4 State Key Laboratory of Respiratory Diseases, Guangzhou Medical University, Guangzhou, People's Republic of China; 5 Department of Microbiology, University of Hong Kong, Hong Kong SAR, China; Duke-NUS Gradute Medical School, Singapore

## Abstract

Human bocavirus (HBoV) is a novel parvovirus associated with respiratory tract diseases and gastrointestinal illness in adult and pediatric patients throughout the world. To investigate the epidemiological and genetic variation of HBoV in Guangzhou, South China, we screened 3460 throat swab samples from 1686 children and 1774 adults with acute respiratory infection symptoms for HBoV between March 2010 and February 2011, and analyzed the complete genome sequence of 2 HBoV strains. Specimens were screened for HBoV by real-time PCR and other 6 common respiratory viruses by RT-PCR or PCR. HBoV was detected in 58 (1.68%) out of 3460 samples, mostly from pediatric patients (52/58) and inpatient children (47/58). Six adult patients were detected as HBoV positive and 5 were emergency cases. Of these HBoV positive cases, 19 (32.76%) had co-pathogens including influenza virus (n = 5), RSV (n = 5), parainfluenza (n = 4), adenovirus (n = 1), coronavirus (n = 7). The complete genome sequences of 2 HBoVs strains (Genbank no. JN794565 and JN794566) were analyzed. Phylogenetic analysis showed that the 2 HBoV strains were HBoV1, and were most genetically close to ST2 (GenBank accession number DQ0000496). Recombination analysis confirmed that HBoV strain GZ9081 was an intra–genotype recombinant strain among HBoV1 variants.

## Introduction

Human bocavirus (HBoV), recently identified as a new member of the Parvoviridae family from the respiratory secretions of children suffering from lower respiratory tract infection [1], has a single-stranded DNA genome of ∼5.2kb, which contains three open reading frames (ORFs), encoding two non-structural proteins (NS1 and NP1) and two viral capsid proteins (VPs) [2]. The nucleotide sequences are highly conserved among HBoVs circulating in different geographic regions [3], with the VP1/VP2 gene displaying relatively commonly found nucleotide polymorphisms [2].

Since its first identification, HBoV has been detected in 1.5%–19% of respiratory tract secretions [1]–[15] and 0.8%–9.1% of fecal samples [13], [16]–[19], respectively, from patients with acute respiratory tract illnesses or gastroenteritis worldwide, It is of note that most of these reports were mainly derived from young children and infants, with only a few exceptions testing adult patients. More recently, three more genotypes of HBoV (HBoV2–4) were found, [2], [20], [21], and reports have shown that inter-genotype and intra-genotype recombinations are present among bocavirus [22]. All 4 genotypes of HBoV have been identified in children with acute gastroenteritis (AGE), whereas only HBoV1 and HBoV2 were reported in respiratory tract samples. Due to the higher rates of co-infections with other pathogens, it remains to be clarified whether in these diseases HBoVs are the key etiologic agent or just a concomitant virus bystander.

To better understand the epidemiology of the HBoV infection, in conjunction of a viral surveillance program, we investigated the presence of HBoV in patients with acute respiratory infection in Guangzhou, a city located in south China. Geographically, the city is characteristics of a tropical-subtropical climate, with the average annual temperature of 20–22°C and average relative humidity of 77%. The city is also highly populated, with a resident population of 12.70 million, plus a non-residential population of 4.76 million. These socio-natural factors make the region generally vulnerable to air-borne as well as food-borne viral infection. The epidemiological status and genomic characteristics of HBoV prevailing in pediatric and adult patients with respiratory infection in the region, however, remains unknown. In our current study, we screened throat swab specimens from patients with acute respiratory tract infection symptoms for HBoV and other common respiratory viruses over a 12-month period using polymerase chain reaction (PCR) methods, and in addition, the molecular phylogeny and complete genome sequences of 2 HBoV strains were also analyzed.

## Materials and Methods

### Ethics statement

All research involving human participants was approved by the Medical Ethics Review Board of Zhongshan School of Medicine, Sun Yat-sen University, in accordance with the guidelines for the protection of human subjects. Written informed consent was obtained from each participant/guardian.

### Patients and specimens

From March 2010 to February 2011, 3460 throat swabs were obtained from 1686 children and 1774 adult patients who had been admitted to five hospitals in Guangzhou, China. They were only taken from individuals with ≤ 3 days of fever (temperature ≥37.5°C), and with cough, sputum, throat sore or other respiratory tract infection symptoms. There were 2009 male and 1451 female patients with age ranging from 1 day to 95 years. Demographic, epidemiology and clinical information including case history, symptoms, physical signs and examination results etc. were collected using a standardized questionnaire. All specimens were added to 2 ml VTM (consists of Earle's Balanced Salt Solution (BioSource International, USA), 4.4% bicarbonate, 5% bovine serum albumin, 100 µg/mL vancomycin, 30 µg/mL amikacin, and 40 U/mL nystatin) according to a standard protocol and transported within 8 hr at 4°C to Biosafety Laboratory of Sun Yat-Sen university, where they were divided into aliquots, and stored at −80°C until processing further.

All specimens were tested for 7 common respiratory viruses, including influenza virus types A, B and C (Inf-A, Inf-B and Inf-C), parainfluenza (PIV) types 1–4, respiratory syncytial virus (RSV), human metapneumovirus (HMPV), human coronavirus (HCoV), adenovirus (AdV) and HBoV using PCR, RT-PCR or real-time PCR methods as described below. Information of patients whose throat swabs were found positive for HBoV was analyzed retrospectively.

### Nucleic Acid Extraction

DNA and RNA were simultaneously extracted from 200 μl of throat swab specimen using QIAamp MiniElute Virus Spin (QIAGEN, Germany). Reverse transcription of virus RNA was conducted by Superscript III transcriptase and random hexamer primers (Invitrogen, Life Technology, USA), both kits were used according to the manufacturer's instructions.

### Pathogen Screening

Inf-A, -B and -C, PIV -1, -2, -3 and -4, RSV-A and -B, HMPV, HCoV, and AdV were detected by a standard reverse transcription-PCR (RT-PCR) or PCR techniques as previously described using specific primers listed in Table S1 [11], [23]–[25], and amplified products were detected by agarose gel electrophoresis.

Screening of HBoV used real-time PCR. The full sequence of HBoV was referred from the ST2 strain (GenBank accession number DQ000496, or NC_007455). TaqMan real-time PCR primers (NP1-F and NP1-R) and probe (synthesized by Invitrogen, Life Technology, USA) were designed to bind the NP1 highly conserved region of different HBoV strains and analyzed by Primer Express software (Version 3.0, Applied Biosystems, USA) (for primer and probe sequences, see Table 1), with regard to optimal G–C content (55%, 44% and 60% for NP1-F, NP1-R and probe, respectively), melting temperature (58.4°C, 59.3°C and 70.0°C for NP1-F, NP1-R and probe, respectively), and amplicon length (88bp). Each reaction mixture consisted of 10 μl 2× iQ Supermix reaction mixture (Bio-Rad, USA), 2 µL of viral DNA, 0.5 µM each of the forward and reverse primers, and 0.3 µM of the probe, and nuclease-free water to a final volume of 20 μl. Real-time PCR was conducted for 95°C for 15 min, followed by 45 cycles of 95°C for 15 s, 60°C for 1 min on the ABI7500 Real-time PCR system.

**Table 1:** The primers used for HBoV screening and complete sequence analysis.

Primer	Sequence (5′–3′)	Position a	Melting Temp.(°C)	PCR product (bp)
NP1-F	AGAGGCTCGGGCTCATATCA	2548–2567	60	88
NP2-R	TCTTCATCACTTGGTCTGAGGTCTT	2635–2611		
HBoV-probe	FAM-AGGAACACCCAATCARCCACCTATCGT-TAMRA	2570–2296		
HBoV-1F	GCCGGCAGACATATTGGATT	1–20	57	722
HBoV-1R	GGACGTGTAGCCAGAAGAGATT	722–701		
HBoV-2F	GGGAGAAGGACTAAGCAAGAG	615–635	52	784
HBoV-2R	GTCAAGCGAAGTAACAGGATG	1398–1378		
HBoV-3F	GGCACGCCTTATAGAACAAGT	1293–1313	56	800
HBoV-3R	GGTAACCACCGCAATCAGT	2092–2074		
HBoV-4F	GAGATTGCAGCTCTTCTACAG	1912–1932	58	729
HBoV-4R	CCTCGTCTTCATCACTTGGT	2640–2621		
HBoV-5F	AAGACAAGCATCGCTCCTAC	2429–2448	55	719
HBoV-5R	ACAGGTTCACCGTTATCAAGT	3147–3127		
HBoV-6F	CACAGACAGAAGCAGACGAGAT	2990–3011	55	776
HBoV-6R	GGTGAGAAGTGACAGCTGTATTG	3765–3743		
HBoV-7F	TGGTCACCTCTACAAAACAGA	3646–3666	55	742
HBoV-7R	GTCTGATGACTGTGGTCCTACT	4387–4366		
HBoV-8F	TCCCAACAAGAAGAGTTCAGT	4242–4262	56.2	1058
HBoV-8R	TGTACAACAACAACACATTAAAAG	5299–5276		

a According to GenBank assession number NC_007455.

### Complete Genome Sequencing for HBoV

The complete genomes of HBoV strains were amplified using primers designed for complete genome by the Primer Premier 5.0 software to bind relatively conserved regions of HBoV as available in the GenBank database (primer sequences shown in Table 1). The PCR was carried out using the Platinum pfx Taq polymerase (Invitrogen, USA) in a prepared reaction mix according to the following condition: 95°C for 5 min, followed by 40 cycles of 94°C for 15 s, 53°C to 58°C (see Table 1 for melting temperature of different primers) for 45 s, and 68°C for 2 min, and a final extension at 72°C for 7 min. PCR products for genome analysis was purified by agarose gel DNA purification kit (Takara, China), and the PCR products of terminal sequences were cloned into PCR-blunt 4-Topo vector (Zero Blunt Topo PCR cloning kit for sequencing, Invitrogen, USA). All PCR products used for cloning and sequencing were from three independent PCR reactions. Sequencing was performed by a commercial service of Invitrogen Co. according to the method described in ref. [26] (Guangzhou, China) and submitted to the GenBank database.

### Phylogenetic Analysis and Recombination Detection

The genomic sequences and ORFs of HBoV were comparatively analyzed with complete genome sequences of other HBoV strains in the GenBank (including HBoV reference strains ST1, ST2, parvovirus B19, bovine parvovirus, canine minute virus, and virus strains obtained from several countries). These sequences were aligned by the Clustal X program, and a neighbor-joining tree was constructed using the MEGA 4.0 software. Potential recombinant sequences and parental sequences were analyzed using the Recombination Detection Program (RDP) [27]. RDP scanning was performed by GENECONV, BOOTSCAN, MaxChi, Chimaera, and SISCAN methods. A Multiple comparison corrected *P*-value cutoff of 0.001 was used throughout. Simplot checking was used to confirm and evaluate localization of possible recombination break points by BOOTSCAN program [28]. Recombinant validation was done by checking the bilateral gene sequences of the recombinant site using phylogenetic trees.

## Results

### Virological Surveillance

Of the 3460 samples collected from patients with respiratory tract infection symptoms and signs enrolled in the study during the period between March 2010 and February 2011, detection for 7 viruses, namely, Influenza, PIV, RSV, HMPV, HCoV, AdV and HBoV, showed that 1275 (36.8%) were found positive for one single virus and 112 (3.2%) were infected by more than one virus. As shown in Table 2, 21.1% of patients tested were found positive for Inf- A, -B, or -C (median age 25 years), 7.7% for RSV (median age 0.91 years), 4.2% for AdV (median age 5 years), 3.5% for PIV (median age 1.2 years), 2.7% for HMPV (median age 2 years), and 2.6% for HCoV (median age 3 years). HBoV DNA was detected in 58 samples (1.68%) (median age 1.5 years) by real-time PCR, including 52 pediatric (47 inpatient, 4 outpatient and 1 emergency patient) and 6 adult (1 inpatient and 5 emergency patients) cases.

**Table 2:** Screening results of respiratory viruses in 3460 patients.

Hospital group	Case number	Positive numbers (infection rate %)						
		Inf	PIV	RSV	HMPV	HCoV	ADV	HBoV
**Outpatient**	**782**	**215**(**27.49**)	**12**(**1.53**)	**26**(**3.32**)	**19**(**2.43**)	**15**(**1.92**)	**29**(**3.71**)	**4**(**0.51**)
Infants	101	11(10.89)	5(4.95)	18(17.82)	1(0.99)	2(1.98)	7(6.93)	4(3.96)
Children	76	14(18.42)	3(3.95)	5(6.58)	2(2.63)	1(1.32)	10(13.16)	0
Adults	605	190(31.40)	4(0.66)	3(0.5)	16(2.64)	12(1.98)	12(1.98)	0
**Emergency case**	**1018**	**366**(**35.95**)	**25**(**2.46**)	**5**(**0.49**)	**8**(**0.79**)	**15**(**1.47**)	**30**(**2.95**)	**6**(**0.59**)
Infants	39	2(5.13)	5(12.82)	4(10.26)	0	1(2.56)	1(2.56)	1(2.56)
Children	83	1(1.2)	7(8.43)	0	0	2(2.41)	9(10.84)	0
Adults	896	363(40.51)	13(1.45)	1(0.11)	8(0.89)	12(1.34)	20(2.23)	5(0.56)
**Inpatient**	**1660**	**150**(**9.04**)	**83**(**5.00**)	**236**(**14.22**)	**67**(**4.04**)	**60**(**3.61**)	**88**(**5.30**)	**48**(**2.89**)
Infants	913	91(9.97)	63(6.9)	203(22.23)	48(5.26)	38(4.16)	33(3.61)	36(3.94)
Children	474	45(9.49)	19(4.01)	24(5.06)	18(3.8)	13(2.74)	36(7.59)	11(2.32)
Adults	273	14 (5.13)	1(0.37)	9(3.3)	1(0.37)	9(3.3)	19(6.96)	1(0.37)
**Total**	**3460**	**731** (**21.13**)	**120** (**3.47**)	**267** (**7.72**)	**94** (**2.72**)	**90** (**2.60**)	**147** (**4.25**)	**58** (**1.68**)
Infants/Children	1686	164 (9.73)	102 (6.05)	254 (15.07)	69 (4.09)	57 (3.38)	96 (5.69)	52 (3.08)
Adults	1774	567 (31.96)	18 (1.01)	13 (0.73)	25 (1.41)	33 (1.86)	51 (2.87)	6 (0.34)

Throat swabs of 3460 patients with acute respiratory infection symptoms during 2010–2011 were collected and screened for influenza (Inf), parainfluenza (PIV), respiratory syncytial virus (RSV), human metapneumovirus (HMPV), human coronaviruses (HCoV), adenovirus (AdV) and human bocavirus (HBoV). Positive case numbers for each screened virus and their corresponding infection rate (% of positive cases in detected patients) were shown, Infants: ≤2 years old, children: 2∼15 years old, adults: >15 years old. Among 731 Inf patients, 558 and 172 were positive for influenza A and B, respectively, but only 1 was positive for C (Fig. S1A). In 558 cases positive for influenza A, 85.13% and 0.72% were identified to be seasonal influenza H3N2 and H1N1, respectively, while 14.15% were found to be 2009 pandemic influenza H1N1. Among 120 cases positive for PIV, 25%, 12.5%, 58.33% and 4.17% were identified to be PIV-1, -2, -3 and -4, respectively (Fig. S1B). Among 90 patients positive for HCoV, 62 cases were identified to be infected by 229E, OC43, NL63 and HKU1, in which infection rates of children vs adults were 0.65% vs 0.17%, 1.13% vs 0.17%, 0.65% vs 0.17% and 0.71% vs 0.06%, respectively.

**Figure 1:**
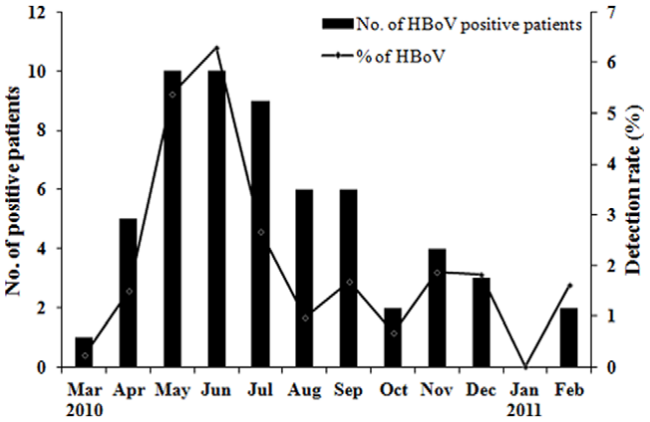
Monthly distribution of HBoV from 3460 patients with acute respiratory infection symptoms during 2010–2011. Human bocavirus (HBoV)-positive case number of each month from March 2010 to February 2011 and the monthly detection rate (% of monthly detected cases) were shown.

The monthly distribution of 7 respiratory viruses tested in patients with indications for respiratory infection from March, 2010 to February, 2011 showed biannual peaks. The highest peak of total positive rate of HBoV and other 6 common respiratory viruses appeared in August (56.2%, chi-square test, *P<*0.05 compared with other months), and another lower peak appeared in winter to spring (January to March). Interestingly, influenza virus was prevalent throughout the year, and peaked in August (Fig. S1). It is also of note that HBoV was detected in nearly all months except January during the study year, and the peak was present in May and June (5.4% and 6.3% respectively, Fig. 1).

**Figure 2:**
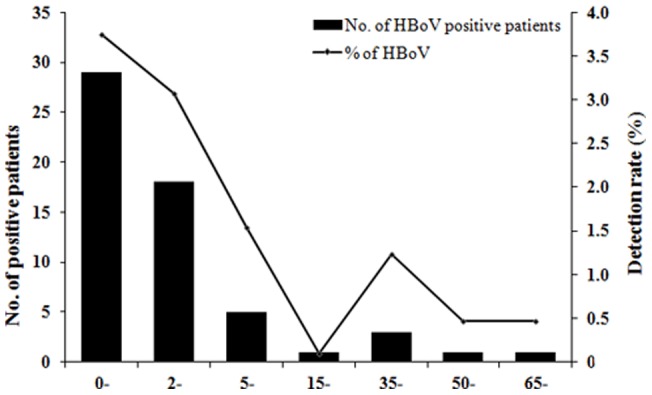
Age distribution of HBoV-positive patients from 3460 patients with acute respiratory infection symptoms during 2010–2011. The number of HBoV-positive patients in different age groups and the corresponding detection rate (% of detected cases in corresponding age group) were shown.

Patients enrolled in this study aged from 1 day to 95 years, including 1686 children (≤15 years old) and 1774 adults (>15 years old) with a median age of 16 years. The total infection rate of common respiratory virus in children is 41.6% (701 positive out of 1686 pediatric subjects), as compared to that of 38.7% (686 positive out of 1774 adult subjects) in adults, For most of the screened respiratory viruses, the infection rate of pediatric patients was higher than adult patients (*P*<0.05) except influenza virus, which tended to infect adults (see Fig. S2 for the age distribution of common respiratory viruses). In contrast to influenza virus, which displayed a higher infection rate (540 out of 731 influenza-infected individuals) in adult patients aged from 15 to 64 years old (Fig. S2), HBoV tended to mostly infect infants younger than 2 years of age (41 out of 58 all HBoV-positive subjects) with a few adult infection (6 in age >15 years old group amongst 58 all HBoV-positive subjects). (Fig. 2).

### Clinical characteristics of HBoV positive cases

In this study, the common symptoms of patients detected as HBoV positive included cough (91.4%), fever (100%),rhinorrhea (36.2%), sputum (36.2%). It is noteworthy that while 43 (74.1%) out of the 58 HBoV-positive patients were clinically diagnosed as lower respiratory tract infection including bronchopneumonia, acute asthmatic bronchopneumonia, and severe pneumonia, only 15 (25.9%) patients met the criteria of severe respiratory infection as featured by dyspnoea. In addition, 9 (15.5%) of the HBoV-positive patients were clinically presented as acute upper respiratory tract infection, and 3 were diagnosed, respectively, as bronchial asthma, herpangina and infectious mononucleosis. Of note, a 77-year-old patient with acute exacerbation of COPD was found infected by HBoV without the presence of any of other 6 respiratory viruses tested in this study. This patient displayed a normal hemogram but a chest radiograph of coarse lung marking.

To better understand the HBoV pathogenicity, clinical characteristics of HBoV-positive outpatients/emergency cases were compared with those of HBoV-positive inpatients (Table 3). The HBoV positive rate in outpatients/emergency was 0.56% (10 out of 1800 outpatients/emergency cases), significantly lower than that of 2.89% in inpatients (48 out of 1660 inpatients) (chi-square test, *P*<0.01). The odds of infection with HBoV resulting in severe disease (or admission) were 5.21 (95% CI 2.64–10.25). HBoV positive pediatric patients were more diagnosed as lower respiratory tract illness than adults in both outpatients and inpatients (3 out of 5 pediatric vs 0 out of 5 adults in outpatient/emergency HBoV-positive patients and 40 out of 47 pediatric vs 0 out of 1 adult in inpatients), especially in infants under 2 years old (35 out of 52 HBoV-positive pediatric patients) (Table 3). In contrast, HBoV positive adults were mainly diagnosed as acute upper respiratory tract infection (AURI) (Table 3). HBoV-positive pediatric inpatients were more likely to be co-infected with other viruses than adult inpatients. However, HBoV-positive adult outpatients were more frequently co-infected with influenza than pediatric outpatients, and the reason may lie in the high influenza infection rate in adults (Table 2 and Table 3).

**Table 3:** Clinical characteristics in HBoV-positive outpatient/emergency and inpatient groups.

Characteristics	Outpatient/emergency		Inpatient		
	Infant/children (%)	Adult(%)	Infant(%)	Children(%)	Adult(%)
Patients, total no.	299	1501	913	474	273
HBoV-positive no.	5	5	36	11	1
HBoV-positive Male/Female	2/3	3/2	26/10	6/5	1/0
HBoV-positive Mean temperature(°C)	38.28	38.86	38.11	37.84	37.5
HBoV-positive patient Diagnosis					
AURI	1(20)	5(100)	2(5.56)	1(9.09)	0
Acute asthmatic bronchopneumonia	0	0	7(19.44)	2(18.18)	0
Bronchopneumonia	3(60)	0	24(66.67)	6(50.45)	0
Severe pneumonia	0	0	1(2.78)	0	0
Bronchial asthma	0	0	1(2.78)	0	0
COPD	0	0	0	0	1(100)
Herpangina	0	0	0	1(9.09)	0
Infectious mononucleosis	0	0	0	1(9.09)	0
HBoV-positive patient Clinical signs					
Cough	5(100)	5(100)	35(97.22)	7(63.64)	1(100)
Rhinorrhea	2(40)	4(80)	12(33.33)	3(27.27)	0
Sputum production	1(20)	1(20)	15(41.67)	3(27.27)	1(100)
Dyspnea	1(20)	0	12(33.33)	1(9.09)	1(100)
Diarrhea	0	0	1(2.78)	0	0
HBoV Copathogens					
Influenza virus	0	3(60)	0	2(18.18)	0
Parainfluenza virus	0	0	4(11.11)	0	0
RSV	0	0	5(13.89)	0	0
Human coronavirus	1(20)	1(20)	3(8.33)	2(18.18)	0
Adenovirus	0	0	1(2.78)	0	0
HBoV Coinfection	1(20)	3(60)	11(30.56)	4(36.36)	0

Of the 3460 cases with acute respiratory infection symptoms, totally 58 cases were detected as HBoV-positive. Because no outpatient/emergency 2∼15 years old children was detected positive for HBoV, there were only 2 columns listed as infant/children and adult in outpatient/emergency patients. HBoV, human bocavirus; RSV, respiratory syncytial virus; AURI, acute upper respiratory tract infection; COPD, chronic obstructive pulmonary disease. Infant: ≤2 years old, children: 2∼15 years old, adults: >15 years old.

### Co-infection

The surveillance data of 7 common respiratory viruses showed that among the 3460 samples, 112 were tested as more than one virus positive. Although influenza was the most common co-infecting virus, the highest co-infection rate occurred in HCoV and HBoV (Table 4). In 19 of 58 HBoV positive specimens (32.76%), other virus can be found, and HCoV was the most commonly co-detected virus with HBoV, accounting for 7 out of 19 (36.84%) HBoV co-infection cases. There were totally 8 triple virus co-infection cases (Table 4). It is noteworthy that despite of the high co-infection rate, in as high as 67.24% HBoV-positive patients, no other screened common respiratory virus was found, especially in pediatric patients (36/52, 69.2%), and most of them (23/36, 63.89%) were diagnosed as bronchopneumonia. No correlation was found between co-infection and clinical symptoms, and among the 19 HBoV co-infection cases, 14 was diagnosed as lower respiratory tract illness, not statistically higher than that of HBoV single positive patients (14/19 vs 29/39, *P*>0.05).

**Table 4:** The co-infection cases of 7 common respiratory viruses including Inf, PIV, RSV, HMPV, HCoV, AdV and HBoV.

Co-detected viruses	Patient No. (%)
Inf, RSV	23 (20.54)
Inf, PIV	9 (8.04)
Inf, HCoV	12 (10.71)
Inf, HMPV	7 (6.25)
Inf, HBoV	4 (3.57)
Inf, AdV	6 (5.36)
PIV, RSV	7 (6.25)
PIV, HCoV	4 (3.57)
PIV, HMPV	3 (2.68)
PIV, HBoV	3 (2.68)
PIV, AdV	2 (1.79)
RSV, HCoV	2 (1.79)
RSV, HMPV	1 (0.89)
RSV, HBoV	3 (2.68)
RSV, AdV	6 (5.36)
HCoV, HMPV	2 (1.79)
HCoV, AdV	4 (3.57)
HCoV, HBoV	5 (4.46)
HBoV, AdV	1 (0.89)
Inf, RSV, HCoV	1 (0.89)
Inf, PIV, AdV	1 (0.89)
Inf, HCoV, HBoV	1 (0.89)
RSV, HCoV, HBoV	1 (0.89)
RSV, HCoV, AdV	1 (0.89)
PIV, RSV, HCoV	1 (0.89)
PIV, RSV, HBoV	1 (0.89)
HCoV, HMPV, AdV	1 (0.89)

Influenza (Inf), parainfluenza (PIV), respiratory syncytial virus (RSV), human metapneumovirus (HMPV), human coronavirus (HCoV), adenovirus (AdV) and human bocavirus (HBoV) were screened during 2010–2011 in Guangzhou, China. Totally 112 cases were detected as more than one virus positive from 3460 patients with acute respiratory infection symptoms. Among them 8 cases were triple virus positive, and 19 cases were co-infection of HBoV and other viruses.

### Sequences and phylogenetic analysis

**Figure 3:**
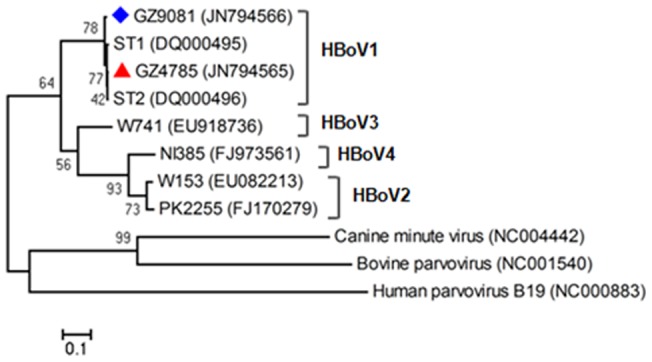
Phylogenetic analysis of human bocavirus based on complete genome. Phylogenetic tree with 1,000 bootstrap replicates was generated using the neighbor-joining method with Mega 4 software. Parvovirus B19, bovine parvovirus, canine minute virus, and representative strains of HBoV1-4 were used as reference strains for genotype analysis of GZ4785 (labeled with red triangle) and GZ9081 (labeled with blue diamond). The HBoV strains obtained in this study GZ4785 and GZ9081 were identified as HBoV1.

**Figure 4:**
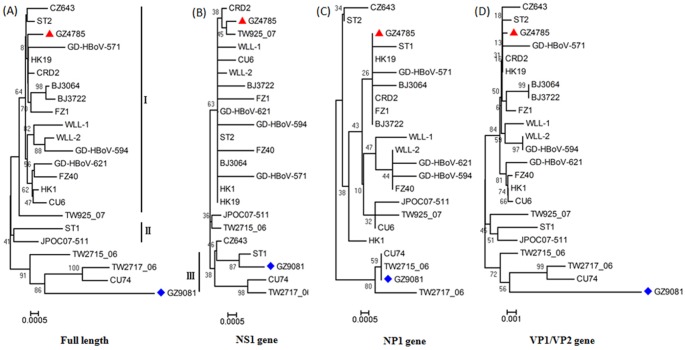
Phylogenetic analysis of GZ4785, GZ9081, and other reference strains based on full length (A), complete NS1 (B), NP1 (C), and VP1/VP2 (D) gene sequences. Phylogenetic trees (1,000 bootstrap replicates, Kimura two-parameter model) based on GZ4785 (labeled with red triangle), GZ9081 (labeled with blue diamond), and other HBoV1 reference strains. Swedish prototype strains ST1 and ST2, American strain CRD2, Japanese strain JPOC07–511, Thai strains CU6 and CU74, Taiwanese strains TW925_07, TW2715_06 and TW2717_06, Chinese strains HK1, HK19, WLL–1, WLL–2, CZ643, FZ1, FZ40, BJ3064, BJ3722, GD-HBoV-571, GD-HBoV-594, GD-HBoV-621 were used (GenBank accession no. DQ000495, DQ000496, DQ340570, AB481080, EF203920, EF203922, EU984245, EU984232, EU984233, EF450717, EF450735, DQ778300, EF441262, DQ457413, GQ455988, GQ455987, DQ988933, DQ988934, GQ926981, GQ926982, GQ926982).

The complete genomes of two HBoV strains GZ4785 (Genbank no. JN794565) and GZ9081 (Genbank no. JN794566) obtained in this study were highly conserved with 98.8% identity to each other, and showed more than 99% nucleotide identity to ST2 strain of HBoV1 (GenBank accession no. DQ0000496). HBoV strains used in the phylogenetic analysis included the strains obtained in this study in Guangzhou (GZ4785 and GZ9081), representative strains of HBoV1-4, human parvovirus B19, bovine, and canine minute virus. Based on complete genome, the phylogenetic analysis results showed that the two Guangzhou strains GZ4785 and GZ9081 were genetically close to HBoV1 (Fig. 3), consistent with the sequence comparison analysis results. From the phylogenetic tree based on complete genome, different strains of HBoV1 were clearly divided into three groups (Fig. 4A), and the representative strain of group I and group II was the prototype strains ST1 and ST2, respectively. Group III included 4 strains which came from Taiwan, Thailand, and Guangzhou. Most Chinese strains obtained from respiratory specimens belonged to group I, but it is noteworthy that GZ9081 strain obtained in this study belonged to group III. No apparently genotypic differences existed between the phylogenetic trees based on the 3 HBoV ORFs (NS1, NP1 and VP1/VP2) and the complete genome of 23 HBoV1 strains (Fig. 4B–D). Similar to previous studies [2], [24], NS1 appeared to be the most conserved gene, whereas VP1/VP2 had the most nucleotide polymorphisms. The phylogenetic trees were almost identical between VP1/VP2 gene and complete genome, which indicated that VP1/VP2 can be used instead of complete genome to analyze the genetical relationship of HBoVs.

### Recombination Detection

**Figure 5:**
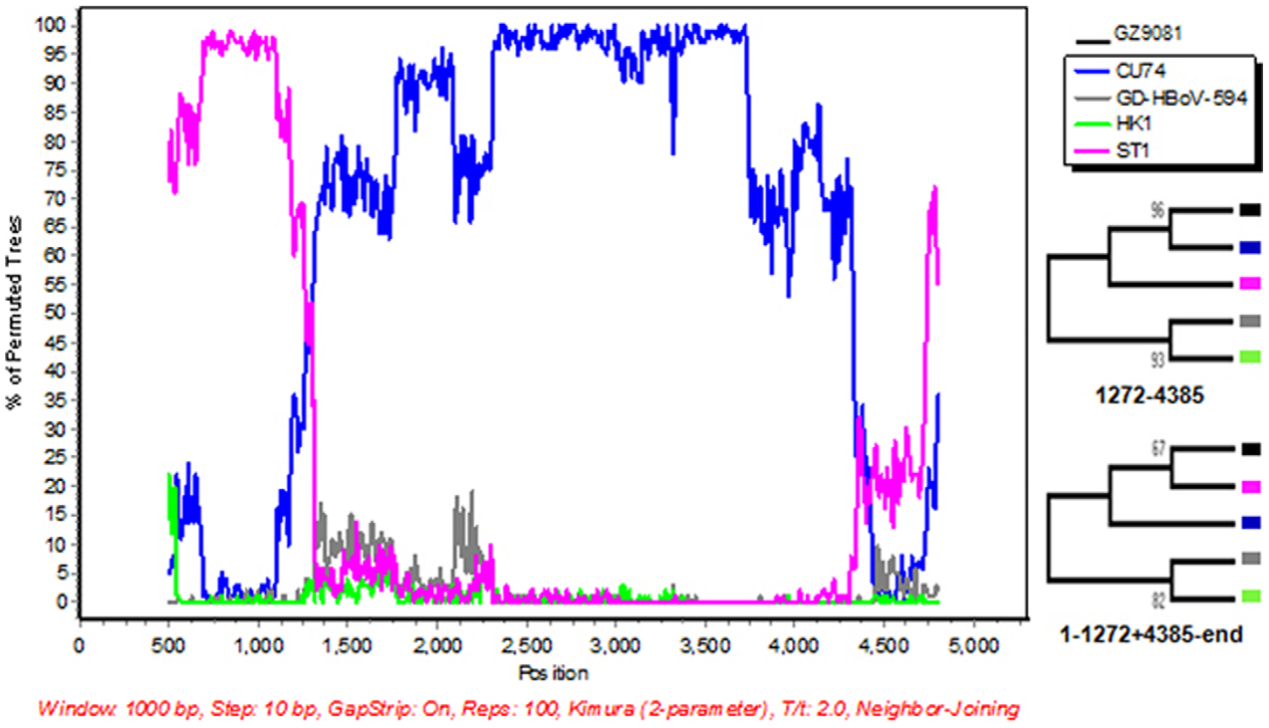
Identification of recombination event between CU74 and ST1, which led to the recombinant strains GZ9081. BOOTSCAN evidence for the recombination origin on the basis of pair-wise distance, modeled with a window size 1000, step size 10, and 100 Bootstrap replicates; The right part of the panel were phylogenetic trees constructed based on recombination regions (1–1272+4385-end) and non-recombination regions (1272–4385) using Mega 4 software.

In phylogenetic analysis, we found that HBoV strain GZ9081 belonged to a group different from most of Chinese strains obtained from respiratory specimens. Interestingly, full-length genome analysis showed that GZ9081 strain contained an NS1 gene closely homologous to that of the ST1 group, and that the rest of its genome (NP1 and VP1/VP2 genes) resembled the CU74 group, suggesting that GZ9081 might represent a hybrid virus, prompting us to conduct the recombinant analysis on this strain. Therefore, RDP was further carried out. The BootScan plot of the recombination event was showed in Fig. 5, which confirmed the daughter linage GZ9081 was a recombinant of parental strains ST1 and CU74. Two trees of the relevant strain were constructed respectively on the recombinant region (position: 1–1272+4385-end) and non- recombinant region (position: 1272–4385), which further confirmed that GZ9081 was closely related with ST1 on the recombinant region (Fig. 5). It was very probable that GZ9081 was an HBoV1 intra-genotypic recombinant.

## Discussion

Human bocavirus (HBoV) was a newly discovered Parvoviridae virus. By now, little is known about its epidemiology and genetic characteristics in Guangzhou, China. The pathogenicity of HBoV is still in uncertain because of its high co-infection rate with other pathogens, and it remains unclear whether HBoVs are sole etiologic agent or just a concomitant virus bystander. Therefore, to understand the prevailing status and pathogenicity of HBoV, other pathogens needed to be simultaneously examined. So in this study, a surveillance of 12 months period during 2010–2011 in Guangzhou was established to understand the prevalence and pathogenicity of HBoV in patients with acute respiratory infection symptoms. Other 6 common respiratory viruses (influenza virus, parainfluenza virus, adenovirus, coronavirus, RSV, metapneumovirus) were screened at the same time to understand their co-infection status with HBoV. The results revealed that the overall monthly distribution of HBoV and other 6 common respiratory viruses were typical, in accordance with the epidemics of respiratory viruses in tropical/subtropical areas like Guangzhou, which demonstrated that the surveillance data in this study were highly reliable. It is notable that HBoV, like RSV, tended to mainly infect ≤2 year old infants, with only a few adult infections, and the majority of the HBoV positive patients (67.24% of all HBoV positive patients and 69.23% of HBoV positive children) were HBoV single positive, indicating it may have potential pathogenicity, especially in infants. Therefore, to better elucidate its pathogenic roles, the clinical characteristics of HBoV-positive outpatients/emergency cases were analyzed for comparison with HBoV-positive inpatients (Table 3). The results showed that the HBoV positive rate in outpatients/emergency was statistically lower than inpatients, and the odds of infection with HBoV resulting in severe disease (or admission) were as high as 5.21. HBoV positive pediatric patients were more diagnosed as lower respiratory tract illness than adults in both inpatients and outpatients, and pediatric inpatients were more likely coinfected with other viruses. Although there was possibility that other co-pathogens not be screened in this study may exist, these results showed that HBoV may have pathogenic role in causing severe disease/admission or lower respiratory tract illness of children. In addition, our results showed that HBoV was related to asthma or its exacerbation, which occurred in 10 pediatric patients.

Previously, only limited data in adults were available for the study of HBoV, especially in large samples. Many reports of adult HBoV infection enrolled no more than 100 samples [2], [8], [12], [29], [30]. The prevalence and associated illness of HBoV in adults have not been well characterized in Guangzhou. In our study, we found 6 HBoV-positive adult cases in 1774 adult patients with upper respiratory tract infection, and the 3.4‰ prevalence rate was in accordance with those previously reported for adults [2], [8], [12], [29], [30]. Although HBoV infection was rare in adults with respiratory infection in our study population, it was still notable that HBoV may cause exacerbation in adults with basic or primary pulmonary disease like COPD. We found 52 HBoV-positive pediatric patients, which represented 3.1% positive rate in pediatric patients with respiratory infection. This is similar to other reports of 1.5%–19%. Consistent with other studies [2], [8], [30], the prevalence rates were higher in children under 2 years of age, but generally decrease with the increase of age (Fig. 2), which implied that antibodies against HBoV acquired during early life may provide protection.

In our study, up to 32.76% co-infection rate of HBoV was observed from throat samples, and the rate may be higher if more viruses were screened. The most frequently detected co-pathogen was HCoV, different from previous reports in China [12], [15], and the reason may lie in different climate/geography conditions and virus distribution. There was no obvious evidence that co-infection of HBoV and other common respiratory viruses can increase the disease severity, since no correlation was found between co-infection and clinical symptoms, and the rate of lower respiratory tract illness did not increase in co-infection cases.

In order to improve the diagnostic sensitivity, our present study employed real-time PCR to screen HBoV with the primers and probes binding the NP1 conserved region. To exclude PCR contamination and prevent false positive and false negative results in HBoV and other 6 common respiratory viruses screening, the following strategies were used. Firstly, all PCR was strictly carried out in 4 separate rooms, namely, reagent preparation, sample preparation, PCR and PCR-product rooms. Secondly, each PCR/real-time PCR assay was performed in duplicates and repeated three times. Thirdly, positive samples were further confirmed by PCR using another set of primers, and for some of the 6 common respiratory viruses positive samples, viral isolation and determination was performed to further confirm the PCR positive results.

Complete genome sequence of 2 HBoV strains were obtained in our study. Gene analysis showed high identity (98.8%) between each other, and phylogenetic analysis demonstrated that they belonged to HBoV1, which was more frequently detected in respiratory tract illness than other genotypes. Phylogenetic tree of complete genome showed that different strains of HBoV1 can be divided into three groups. It is noteworthy that the two strains identified in this study (GZ4785 and GZ9081), which were circulating at the same time during the 1 year study period, belonged to different groups. Furthermore, our phylogenetic analysis results showed that most Chinese HBoV1 strains obtained from respiratory samples belonged to group I which was genetically closely to ST2, but GZ9081 was different, which belonged to group III. Phylogenetic trees based on different HBoV ORFs showed that its NS1 gene was closely homologous to the ST1 group, whereas its NP1 and VP1/VP2 genes resembled the CU74 group, suggesting it may be a recombinant. Although generally HBoV1 sequences were highly conserved, there were still evidences that the recombination existed among HBoVs [22], and co-infection of HBoVs [20] might increase the chance of recombination between HBoVs. Therefore, we suspect that GZ9081 may be a hybrid virus. So a recombinant detection program was performed to analyze GZ9081. The result confirmed that GZ9081 was an intra-genotype of HBoV1 recombinant, originated from the parental strains ST1 and CU74 (Fig. 5). As far as we know, this is the first time that recombination between HBoV1 is reported and this is the first recombinant strain of HBoV1 reported in China. Since recombination could change virulence or antigenicity of viruses, we believe that the finding of HBoV recombination might have significance on the epidemiology, seroprotection and pathogenicity study of HBoV. Further studies are needed to examine the virulence and antigenic changes of GZ9081 strain. Continuous surveillance and genome sequence analysis are needed to obtain more information on the genotypic variation and molecular evolution of HBoV in China.

## Supporting Information

Figure S1 **Monthly distribution of 6 common respiratory viruses from 3460 patients with acute respiratory infection symptoms from March 2010 to February 2011.** Virus-positive case number of each month and the monthly detection rate (% of monthly detected cases) were shown. (A) inﬂuenza virus (Inf); (B) parainﬂuenza virus (PIV); (C) respiratory syncytial virus (RSV); (D) adenovirus (AdV); (E) human metapneumovirus (HMPV); (F) human coronavirus (HCoV).
(TIF)

Figure S2 **Age distribution of 6 common respiratory viruses from 3460 patients with acute respiratory infection symptoms from March 2010 to February 2011.** The number of virus-positive patients of different age groups, and the corresponding detection rate (% of detected cases in corresponding age group) were shown. (A) inﬂuenza virus (Inf); (B) parainﬂuenza virus (PIV); (C) respiratory syncytial virus (RSV); (D) adenovirus (AdV); (E) human metapneumovirus (HMPV); (F) human coronavirus (HCoV).
(TIF)

Table S1 **The primers used for Inf, PIV, RSV, HMPV, HCoV and AdV screening.** Inf: Influenza, PIV: parainfluenza, RSV: respiratory syncytial virus, HMPV: human metapneumovirus, HCoV: human coronavirus, AdV: adenovirus.
(DOC)
