# Supplementary figures and images for: Surveillance and Genome Analysis of Human Bocavirus in Patients with Respiratory Infection in Guangzhou, China
Source: PLoS One. 2012 Sep 11;7(9):e44876. doi: 10.1371/journal.pone.0044876 (PMC3439446; doi:10.1371/journal.pone.0044876)

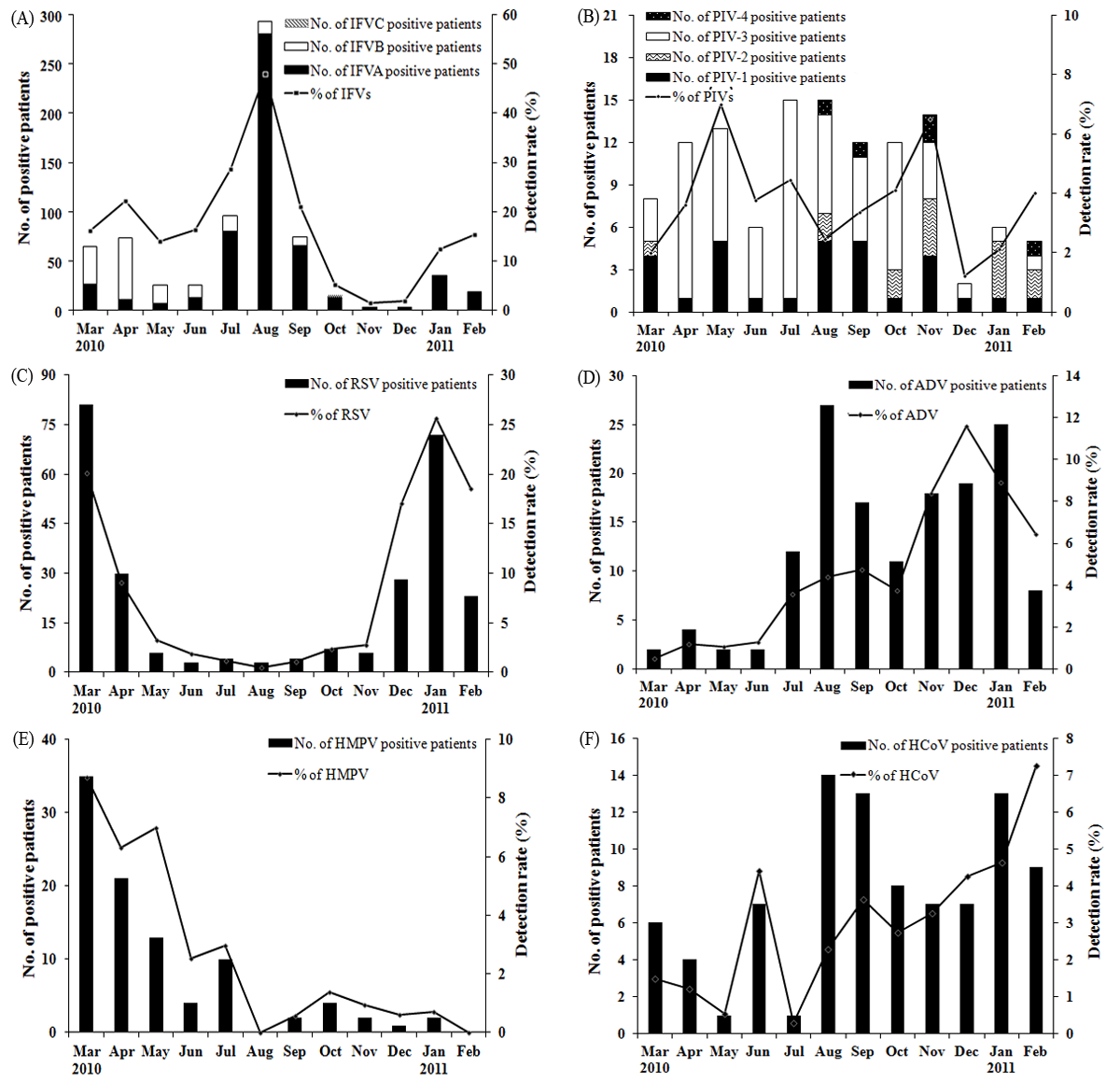

Supplement: Figure S1 — Monthly distribution of 6 common respiratory viruses from 3460 patients with acute respiratory infection symptoms from March 2010 to February 2011. Virus-positive case number of each month and the monthly detection rate (% of monthly detected cases) were shown. (A) inﬂuenza virus (Inf); (B) parainﬂuenza virus (PIV); (C) respiratory syncytial virus (RSV); (D) adenovirus (AdV); (E) human metapneumovirus (HMPV); (F) human coronavirus (HCoV). (TIF) [file pone.0044876.s001.tif]

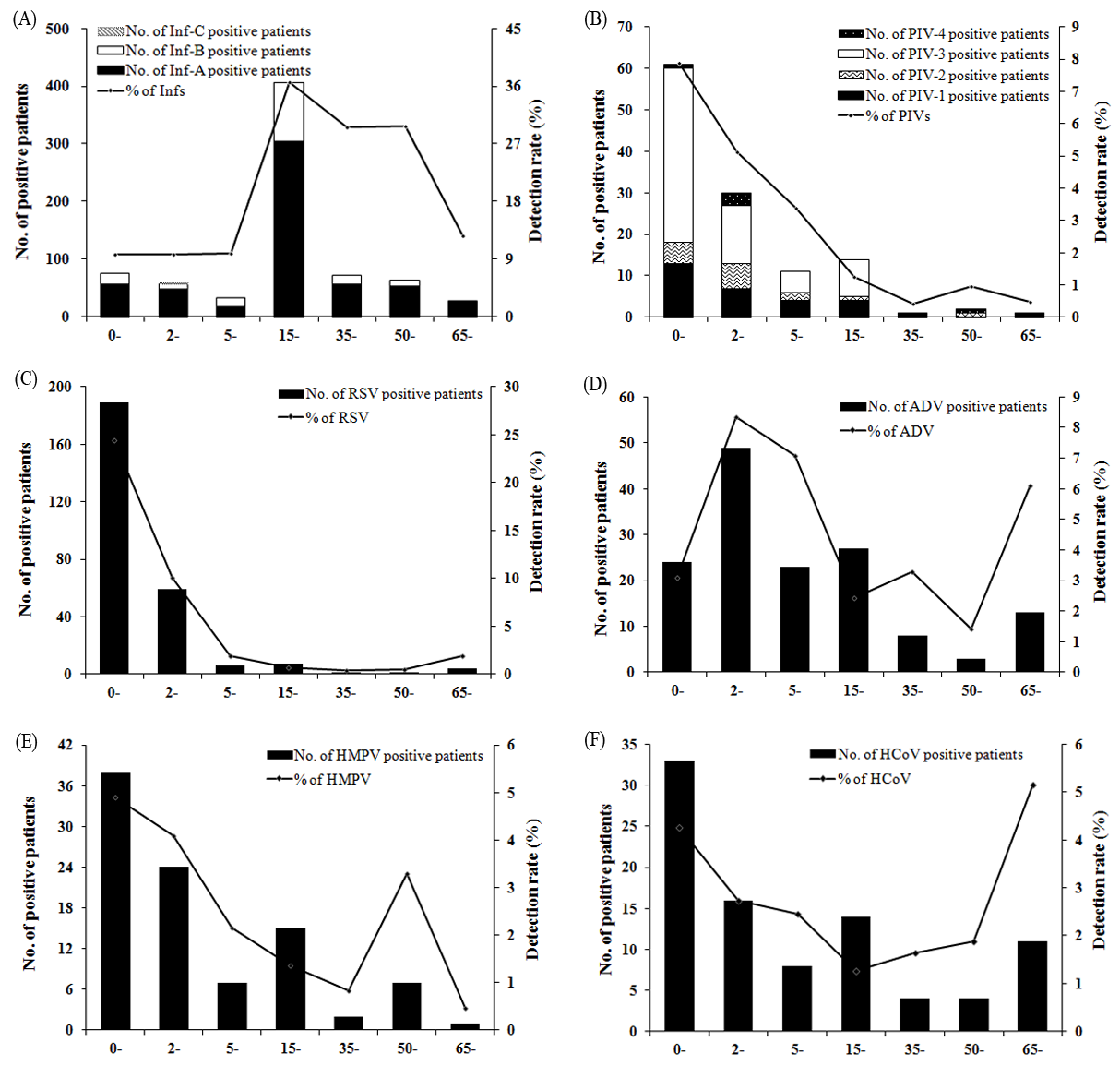

Supplement: Figure S2 — Age distribution of 6 common respiratory viruses from 3460 patients with acute respiratory infection symptoms from March 2010 to February 2011. The number of virus-positive patients of different age groups, and the corresponding detection rate (% of detected cases in corresponding age group) were shown. (A) inﬂuenza virus (Inf); (B) parainﬂuenza virus (PIV); (C) respiratory syncytial virus (RSV); (D) adenovirus (AdV); (E) human metapneumovirus (HMPV); (F) human coronavirus (HCoV). (TIF) [file pone.0044876.s002.tif]
