# Supplementary material for: Surveillance and Genome Analysis of Human Bocavirus in Patients with Respiratory Infection in Guangzhou, China
Source: PLoS One. 2012 Sep 11;7(9):e44876. doi: 10.1371/journal.pone.0044876 (PMC3439446; doi:10.1371/journal.pone.0044876)
Supplement: Table S1 — The primers used for Inf, PIV, RSV, HMPV, HCoV and AdV screening. Inf: Influenza, PIV: parainfluenza, RSV: respiratory syncytial virus, HMPV: human metapneumovirus, HCoV: human coronavirus, AdV: adenovirus. (DOC) [file pone.0044876.s003.doc]

**Table S1 The primers used for Inf, PIV, RSV, HMPV, HCoV and AdV screening**

| **Virus** | **Primer** | **Sequence（5’-3’）** | **Position** | **PCR product (bp)** |
| --- | --- | --- | --- | --- |
| Inf, RSV  First round | FluAC-F1 | GAACTCRTYCYWWATSWCAAWGRRGAAAT | NP |  |
|  | FluB-F1 | ACAGAGATAAAGAAGAGCGTCTACAA |  |  |
|  | FluABC-R1 | ATKGCGCWYRAYAMWCTYARRTCTTCAWAIGC |  |  |
|  | RSVAB-F1 | ATGGAGYTGCYRATCCWCARRRCAARTGCAAT | F |  |
|  | RSVAB-R1 | AGGTGTWGTTACCCTGCATTRACACTRAATTC |  |  |
| Inf, RSV  Nested PCR | FluAB-F2 | GATCAAGTGAKMGRRAGYMGRAAYCCAGG | NP | (Inf-A)301  (Inf-B)226  (Inf-C)111 |
|  | FluC-F2 | AAATTGGAATTTGTTCCTTTCAAGGGACA |  |  |
|  | FluAC-R2 | TCTTCAWATGCARSWSMAWKGCATGCCATC |  |  |
|  | FluB-R2 | CTTAATATGGAAACAGGTGTTGCCATATT |  |  |
|  | RSVA-F2 | TTATACACTCAACAATRCCAAAAAWACC | F | RSV  (A)363  (B)611 |
|  | RSVA-R2 | AAATTCCCTGGTAATCTCTAGTAGTAGTCTGT |  |  |
|  | RSVB- F2 | ATCTTCCTAACTCTTGCTRTTAATGCATTG |  |  |
|  | RSVB- R2 | GATGCGACAGCTCTGTTGATTTACTATG |  |  |
| PIV  First round | PIV13-F | AGGWTGYSMRGATATAGGRAARTCAT | HA | PIV1：439  PIV2：297  PIV3：390  PIV4：174 |
|  | PIV13-R | CTWGTATATATATRTAGATCTKTTRCCTAGT |  |  |
|  | PIV2-F | TAATTCCTCTTAAAATTGACAGTATCGA |  |  |
|  | PIV4-F | ATCCAGARRGACGTCACATCAACTCAT |  |  |
|  | PIV24-R | TRAGRCCMCCATAYAMRGGAAATA |  |  |
| PIV  Nested PCR | PIV13-F | ACGACAAYAGGAARTCATGYTCT |  |  |
|  | PIV1-R | GACAACAATCTTTGGCCTATCAGATA |  |  |
|  | PIV3-R | GAGTTGACCATCCTYCTRTCTGAAAAC |  |  |
|  | PIV24-F | CYMAYGGRTGYAYTMGAATWCCATCATT |  |  |
|  | PIV2-R | GCTAGATCAGTTGTGGCATAATCT |  |  |
|  | PIV4-R | TGACTATRCTCGACYTTRAAATAAGG |  |  |
| HCoV | hCoV-F | GGTTGGGACTATCCTAAGTGTGA | POL | 440 |
|  | hCoV-R | CCATCATCAGATAGAATCATCATA |  |  |
| HMPV | hMPV-F | CATGCCCACTATAAAAGGTCAG | L | 171 |
|  | hMPV-R | CACCCCAGTCTTTCTTGAAA |  |  |
| ADV | ADV-F | GCCSCARTGGKCWTACATGCAC ATC | Hexon | 301 |
|  | ADV-R | CAGCACSCCICGRATGTCAAA |  |  |
